# Frailty Development in Older Adults: Insights from an Eight-Year Longitudinal Study

**DOI:** 10.1093/geroni/igaf122.2369

**Published:** 2025-12-31

**Authors:** Khalil Iktilat, Aviad Tur-Sinai, Netta Bentur, Netta Bentur, Debbie Rand

**Affiliations:** Tel Aviv University, Tel Aviv, Tel Aviv, Israel; University of Haifa, Haifa, HaZafon, Israel; Tel Aviv University, Tel Aviv, Tel Aviv, Israel; Tel Aviv University, Tel Aviv, Tel Aviv, Israel

## Abstract

Frailty is a key marker of biological aging, associated with adverse health outcomes and loss of independence. Most studies focus on risk factors using cross-sectional data, limiting understanding of individual transitions. This study utilizes an eight-year longitudinal follow-up across European countries to assess how demographic, socioeconomic, and health factors influence frailty progression. Data of adults aged 65–84 were drawn from Waves 4-8 (2011-2020) of the SHARE survey, across 27 countries. Frailty was assessed using Fried’s criteria (grip strength, walking speed, weight loss, exhaustion, and physical activity). Participants were classified as non-frail, pre-frail, or frail based on cumulative deficits in these domains. Key predictors included age, sex, chronic conditions, socioeconomic status, self-rated health, and physical activity levels. Frailty progression followed a cumulative pattern. Over two years, 65.96% of 18,331 participants remained non-frail, 32.10% became pre-frail, and 1.94% transitioned to frailty. By eight years, only 57.44% remained non-frail, while 37.55% became pre-frail and 5.02% developed frailty. Older age (OR = 1.098, p < 0.001) and poor self-rated health (OR = 0.751, p < 0.001) were the strongest predictors. Physical activity was protective, particularly indoor (OR = 1.294, p < 0.001) and outdoor activity (OR = 1.157, p = 0.019), while financial distress (OR = 1.143, p = 0.005) increased frailty risk. These findings emphasize the high risk of frailty progression in older adults without intervention, leading to increased health deterioration. Identifying key risk factors can guide targeted strategies to slow frailty onset and reduce its impact. Promoting physical activity, social engagement, and financial stability may help delay frailty progression and improve health outcomes in aging populations.

